# New insights into methods to measure biological age: a literature review

**DOI:** 10.3389/fragi.2024.1395649

**Published:** 2024-12-16

**Authors:** Aanchal Mathur, Sebastien Taurin, Sfoug Alshammary

**Affiliations:** Department of Molecular Medicine, Princess Al Jawhara Center, College of Medicine and Health Sciences, Arabian Gulf University, Manama, Bahrain

**Keywords:** biological age, epigenetic age, exosomes, microbiome, chronologic age

## Abstract

Biological age is a concept that reflects the physiological state of an individual rather than the chronological time since birth. It can help assess the risk of age-related diseases and mortality and the effects of interventions to slow down or reverse aging. However, there is no consensus on measuring biological age best, and different methods may yield different results. In this paper, which includes 140 relevant pieces of literature, out of 33,000, we review some new methods to measure biological age based on recent advances in biotechnology and data science. We discussed some novel biomarkers and algorithms that can capture the dynamic and multidimensional aspects of aging at different levels. We evaluate their performance and validity using various datasets and criteria and compare them with existing methods. We also discuss their potential applications and implications for aging research and clinical practice. We conclude that the new methods offer more accurate and reliable estimates of biological age and open new avenues for understanding and modulating the aging process.

## 1 Introduction

Aging can be defined as the time-related deterioration of the physiological functions necessary for survival and fertility. It is a gradual, continuous process of natural change that begins in early adulthood and affects all individuals of a species. Aging is caused by accumulating a wide variety of molecular and cellular damage over time, leading to a gradual decrease in physical and mental capacity, a growing risk of disease, and, ultimately, death. According to the World Health Organization (WHO), the world’s population of people aged 60 years and older will double by 2050, reaching 2.1 billion. By 2030, 1 in 6 people in the world will be aged 60 years or over. For the longest time, age has only been calculated using the chronological measure of aging (Jia et al., 2017). Traditionally, chronological age has been defined as the period elapsed since an individual’s birth (Yaneske and Angione, 2018). In contrast, biological age is an alternative measure depending on the molecular damage the body accumulates over time (Beltrán-Sánchez et al., 2022). It is typical for the chronological and biological ages to differ (Maltoni et al., 2022). Lately, biological age has been preferentially used over chronological age, as it offers a more pertinent evaluation of an individual health span and lifespan. It captures genetic, metabolic, and environmental changes experienced by an individual. Biological aging is, therefore, a more effective measure of an individual’s health span and lifespan (Jazwinski and Kim, 2019).

The collection of twelve interdependent indicators recently reviewed by López-Otín et al. (2023) provides a fundamental framework for deciphering the evolution of aging. These hallmarks are inclusive of elements such as genomic instability, telomere attrition, epigenetic alterations, decline in proteostasis, impaired macro-autophagy, nutrient-sensing deregulation, mitochondrial dysfunction, cellular senescence, stem cell exhaustion, altered intercellular communication, chronic inflammation, and dysbiosis (López-Otín et al., 2023)The recently identified hallmarks of aging coincide with and are interconnected with the hallmarks of health. These newly recognized factors intersect and intertwine with essential facets of health. An individual’s progress in age can be measured depending on the combination of primary, secondary, or tertiary hallmarks the person possesses (Gems and de Magalhães, 2021).

In the exploration of biological age, telomere shortening, and epigenetic clocks are the most common methods. Telomeres, the chromosomal extremities that gradually reduce in length as cellular division proceeds, have an integrative role in safeguarding overall cellular health. Telomere shortening is associated with a plethora of health conditions, with telomere lengths exhibiting plasticity in response to socio-environmental variables (Ledda et al., 2020). Epigenetic clocks are built on a collection of CpG sites whose methylation levels correlate to an individual’s chronological age (Field et al., 2018). They have emerged as a highly accurate molecular parallel of human and other vertebrates’ chronological age. These epigenetic clocks have recently been utilized to measure and study biological aging (Huang et al., 2022). Aging-associated alterations in DNA methylation patterns have suggested an intrinsic mechanism that drives human aging (Joseph et al., 2018). The epigenetic clocks have yet to reach the telomere length’s credibility for determining aging and overall lifespan (Vaiserman and Krasnienkov, 2020). The role of environmental factors and life experiences in shaping our epigenome over time is well recognized (Toraño et al., 2016). However, the formulation of epigenetic clocks employs linear regression, presuming a linear interplay between DNA methylation and age. This assumption, however, may lack potency across all CpG sites, and thus, the model’s accuracy could vary among individuals (Kabacik et al., 2022). Given the ongoing debate around a gold standard for the measurement of biological age, the pursuit of advanced methodologies continues within the scientific community.

This review discusses the contemporary strategies for assessing biological aging alongside mentioning some non-traditional measurement methods. It investigates the key indicators of aging, in pursuit of establishing a benchmark for the quantification of biological age.

## 2 Methodology

**FIGURE 1:**
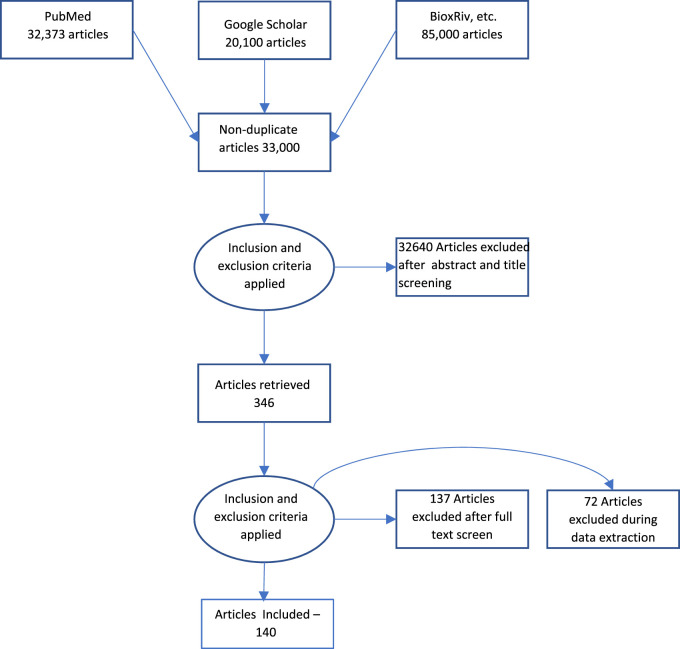
Prisma Flow diagram of search methodology.

Acomprehensive literature search was performed using MEDLINE/Pubmed and Google Scholar as indicated in Figure 1. The keywords used were “Measurement,” AND “Biological Aging” AND “telomere length”, “epigenetic clock”, “epigenetic aging”, “genomic instability”, “hallmarks”, “aging”, “cellular senescence”, “mitochondrial dysfunction”, “mitochondrial aging,” “stem cell aging”, “stem cell”, “microbiome”, “gut microbiome”, “microbiome age”, “exosome”, “exosome aging”, “biological age”, “chronological age”. The articles included were dated from January 2019 to September 2023. The search result yielded an approximate total of 33,000 articles across all keyword searches. The articles were further filtered based on relevance to the review as well as repetition. The final result yielded approximately 140 articles, which have been referenced in this review.

## 3 Mechanism of aging

Aging is a complex process characterized by accumulating disrepairs within the organism, induced by intrinsic and extrinsic stresses (Guo et al., 2022). An array of repair mechanisms typically counteracts these stressors. However, when these mechanisms fail to function correctly, it leads to escalated molecular injuries, inflammation, and flawed cellular signaling. These factors contribute to random molecular damage, cellular defects, and tissue dysfunction, all of which contribute to aging (Zhao et al., 2023). Intrinsic factors for aging could be the accumulation of reactive oxygen species and reactive nitrogen species caused by inflammation and stress on cells (Warraich et al., 2020). Extrinsic factors could be environmental toxins, UV radiation, diet, and sedentary lifestyle (Franceschi et al., 2018). The exposure of the body to stressors and damage-causing environments can cause genetic and epigenetic changes to occur (Dee et al., 2023). These may lead to irreversible cellular senescence, telomere shortening, and mutations.

The accumulation of damaged proteins can occur with the downregulation of transcription factors, such as Heat shock factor-1 (HSF-1) and FOXO family ortholog (DAF-16), which downregulate heat shock proteins (HSP), thereby causing errors in protein folding (Kikis et al., 2010). Researchers have identified that upregulation of HSP by increased activity of the Hsp70 gene can increase lifespan (Radons, 2016). As the individual ages, the accumulated damage leads to the dysfunction of autophagy and lysosomal systems, further causing protein degradation due to the accumulation of free radicals (Pajares et al., 2018). The ubiquitin-proteosome system also accumulates damage over time, leading to aging. Upregulation of epidermal growth factor and activation of protein catabolism delay aging.

Accumulation of mutations and DNA damage is one of the most common causes of aging (World Health Organization, 2021). This could include insertions, deletions, and miss-match. Two other commonly studied causes of aging are telomere shortening, leading to low cell proliferation and cellular senescence, the other is epigenetic alterations such as histone modification, chromatin remodeling, and DNA methylation, which lead to age-related diseases and memory defects. Progeria is an ideal example of the effect of mutations in the genes. Damage to the mitochondrial DNA and mutations to this DNA has been found in people with age-related disorders such as Alzheimer’s disease, coronary artery disease, Parkinson’s disease, etc.

## 4 Conventional measures of biological aging

### 4.1 Genomic instability

Genomic instability is linked to aging and can occur due to exogenous or endogenous means. In a healthy body, the DNA repair mechanism heals the damage caused by these exogenous and endogenous components (Niedernhofer et al., 2018). Over time as the body age, this repair mechanism declines, causing an accumulation of reactive oxygen species and DNA mutations and further leading to miss-matched proteins and loss of function. Genomic instability is generated when any one tier of the DNA repair mechanism fails; tier 1 removal of reactive oxygen species, tier 2 conserved repair mechanisms such as direct reversal, base excision repair, nucleotide excision repair, double-strand break repair, and inter-strand crosslink repair, and tier 3 activation of programmed cell death, senescence, and cell cycle arrest (Chatterjee and Walker, 2017). As these mechanisms occur over the lifetime of individuals, multiple factors can lead to disruption in the repair mechanism and cause age-related disease.

Multiple genes have been identified and linked to longevity. These genes include FOXO3A, APOE, TERT, SOD1, and IGF1R. The FOXO3A gene encodes a transcription factor that plays a role in cell survival, metabolism, and stress resistance (Davy et al., 2018). Mutations in FOXO3A have been linked to premature aging syndromes (Davy et al., 2018). The APOE gene encodes a protein that transports cholesterol in the blood. Certain variants of APOE have been associated with an increased risk of Alzheimer’s disease and other age-related diseases (Raulin et al., 2022). TERT gene encodes the telomerase reverse transcriptase enzyme, which is responsible for maintaining telomere length. Telomeres are protective caps at the ends of chromosomes that shorten with each cell division (Leão et al., 2018). Shorter telomeres have been linked to an increased risk of age-related diseases and mortality (Gruber et al., 2021). Similarly, the SOD1 gene encodes the superoxide dismutase 1 enzyme, which helps to protect cells from damage caused by free radicals. Mutations in SOD1 have been linked to amyotrophic lateral sclerosis (ALS), a progressive neurodegenerative disease (Wang et al., 2018). Finally, the IGF1R gene encodes the insulin-like growth factor 1 receptor, which plays a role in growth, metabolism, and aging. Mutations in IGF1R have been linked to increased longevity (Vitale et al., 2019). Other genes, such as CISD2, SIRT1, and SIRT2, have recently been discovered to be associated with longevity in humans. These genes are commonly found in centenarians in many parts of the world (Ferrucci et al., 2020). SIRT1 is located on chromosome 10q21.3, indicating its cardioprotective effects. It is an NAD + -dependent histone deacetylase that acts as a transcription factor and a cofactor in addition to being a target for histone and non-histone proteins (Askin et al., 2020). SIRT2 is a member of the Sirtuin family and is the only cytoplasmic member of the family (Yang et al., 2023). Both these genes, while associated with longevity, cannot be measured to determine an individual’s biological age. Not much is known about their pathways and proteins that slow down aging. Although mutations in the form of insertions, deletions, and translocations have been studied in association with age-related disorders, much research is yet to be done to utilize genomic instability as a measure of biological age.

### 4.2 Telomere length

Telomeres are susceptible to damage in the DNA and shorten exceptionally whenever a DNA stressor is available. Their damage is associated with genomic instability and cellular senescence, making telomere attrition a primary hallmark of aging (De Meyer et al., 2018). Deterioration in the telomeres is challenging to repair by the body’s repair mechanisms. This is because oxidative stress causing free radicals inhibit the function of telomerase and cause telomere shortening (Barnes et al., 2019). The reduction of telomerase also causes disruption in telomere-binding proteins and leads to uncapping of DNA by the telomeres (Morgan et al., 2018). Telomeres play a vital role in protecting the integrity of the genome from nucleolytic degradation, DNA damage response, and unnecessary recombination. They comprise multiple kilobases of G-rich tandem repeat DNAsequencesending with a 50–400 nucleotide single-stranded 3′overhang and organized in a peculiar chromatin structure (Oblak et al., 2021). In humans, the chromatin structure of telomeres involves the shelterin protein complex and the noncoding RNA TERRA (Lim and Cech, 2021). The shelterin complex comprises six proteins, including telomeric repeat binding factor 1 (TRF1, encoded by the TERF1 gene), telomeric repeat-binding factor 2 (TRF2), TPP1 (or ACD,recruiting telomerase), protection of telomeres 1 (POT1, encoded by POT1), TRF1 interacting nuclear factor 2 (TIN2, encoded by TINF2), and TRF2 interacting protein (RAP1 orTERF2IP) (Veverka et al., 2019). These factors have multiple functions, such as telomere replication regulation, capping, and higher-order structure determination of telomeric chromatin. Uncapping shelterin complexes from telomeres leads to the activation of the DNA damage response, further leading to DNA repair at telomeres. Shelterin deficiency leads to telomere uncapping and even telomere collapse (Mir et al., 2020).

In humans, the telomere length at birth consists of short tandem repeats (5′-TTAGGG-3′) that range from 8 to 15 kb (Srinivas et al., 2020). Over time during development, telomere DNA is elongated by telomerase to counteract dramatic telomere shortening by 50–200 nucleotides after each replication cycle (Sanford et al., 2020). This occurs due to the increased proliferation of cells at this stage of development. Further, telomere length acts as a counter for the number of cellular divisions during development. Programmed telomere shortening, which leads to the threshold telomere length (the Hayflick limit), triggers replicative senescence (irreversible cell cycle arrest) (Lupatov and Yarygin, 2022). This makes telomere length an excellent biomarker to measure biological aging. The most commonly used methods to detect or measure the telomere length are quantitative polymerase chain reaction (Q-PCR), terminal restriction fragment (TRF) analysis, a variety of quantitative fluorescence *in situ* hybridization (Q-FISH) methods, single telomere length analysis (STELA), telomere shortest length assay (TeSLA), peptide nucleic acid hybridization analysis of single telomere (PHAST) assay, and single-molecule real-time (SMRT) sequencing (Lai et al., 2018). Telomere length cannot be considered a gold measurement standard as it is not a linear measure of aging (Srinivas et al., 2020). Telomere length shortens at a constant rate in early life, but the rate of shortening slows down in later life (Whittemore et al., 2019). It can therefore provide a false age in the case of older populations. Telomere length is also affected by several factors other than aging (Andreu-Sánchez et al., 2022). These factors include genetics, lifestyle, and environmental exposures. This means that telomere length can be a poor predictor of aging in individuals exposed to these factors. Furthermore, there is no agreement on the optimal method for measuring telomere length, indicating that results from different studies may not be comparable.

### 4.3 Cellular senescence

Cellular senescence is a biological measure of aging that refers to a state of stable cell cycle arrest in which proliferating cells become resistant to growth-promoting stimuli (Huang et al., 2022). It is considered a hallmark of aging and linked to aging-related diseases (López-Otín et al., 2013). Senescent cells accumulate with age and contribute to age-related disorders such as cancer, neurodegeneration, and other age-related pathologies (Wissler Gerdes et al., 2020). Hayflick and Moorhead first identified this form of replicative arrest in the 1960s on a fibroblast cell which showed permanent cell cycle arrest (Alspach et al., 2013). Senescence can occur as a manifestation of multiple hallmarks of aging. The most common being telomere attrition caused due the over-expression of senescent genes and lack of shelterin genes (POT1 or TRF), leading to the uncapping of free double-stranded chromosome end and triggering a permanent DNA damage response (Jebaraj and Stilgenbauer, 2020). Other factors that cause cellular senescence may be by mitogenic signals, oncogenic activation, radiation, oxidative and genotoxic stress, epigenetic changes, chromatin disorganization, perturbed proteostasis, mitochondrial dysfunction, inflammation, and tissue damage signals, irradiation, or chemotherapeutic agents, nutrient deprivation (Kumari and Jat, 2021). These factors generate signals that are identified by the body as stress signals and can give rise to different types of induced cell death. Cellular senescence can be telomere-dependent replicative senescence, programmed senescence, or non-telomeric stress-induced premature senescence, including oncogene-induced senescence (OIS), unresolved DNA damage-induced senescence, epigenetically induced senescence, and mitochondrial dysfunction associated senescence (Nousis et al., 2023). In all cases, two main pathways significantly initiate senescence: the p53/p21CIP1 pathway and the p16^INK4a^/RB pathway (Mijit et al., 2020). Stress signals caused by telomere arritition and oxidative stress generate signals activating the overexpression of p53 induced by miRNA (miR-504) and lncRNAs, ubiquitination (MDM2 ligase and MDMX), and binding of FOXO4 to p53 (Liu and Xu, 2011). On the other hand, p21, encoded by the CDKN1Agene, causes cell cycle arrest at G1/S and G2/M checkpoints by suppressing cyclin E-CDK2 and cyclin A-CDK2 complex formation (Al Bitar and Gali-Muhtasib, 2019). This leads to a p53-induced cell cycle suspension at the G1 phase. p21 triggered senescence can also be activated by TGF-β/SMAD and PI3K/FOXO pathways, independent of p53, and can play a crucial role in transient programmed cellular senescence during embryonic development (Liao et al., 2021). Transcription factors such as Sp1, Ets, AP1 (JunB subunit), PPARγ, HBP-1, CTCF, and FOXA1 activate INK4a encoded p16 expression (Rayess et al., 2012a). Similarly, ITSE (INK4a transcription silence element), YB1, ID1, and AP-1 (c-Jun subunit) reduce p16INK4a expression leading to cellular senescence caused by external or internal stimuli (Li et al., 2011). p16 inhibits the formation of CDK4/6 complexes, preventing phosphorylation of RB and promoting the construction of the RB–E2F complex (Rayess et al., 2012b). This leads to the inhibition of cell-cycle genes. Multiple upstream and downstream factors regulate these complex pathways but lead to age-related sustained cellular senescence. SABG, p21, and p16 are the most commonly used senescence markers in addition to DNA replication markers EdU or BrdU, cell proliferation markers Ki-67/ PCNA, DNA damage markers γH2AX, Lamin B1, and SASP markers (Wagner and Wagner, 2022). These markers are visualized using flow cytometry, Q-PCR, and fluorescence *in situ* hybridization. Prolonged cellular senescence caused by accumulated damage over time can lead to multiple disorders such as chronic kidney disease, cancer, osteoporosis, metabolic syndrome, type 2 diabetes mellitus, reproductive aging, atherosclerosis, neurodegeneration, and glaucoma (Huang et al., 2022). Currently, senolytics are being used to treat cellular senescence-related aging disorders. Still, more studies need to be done to identify the effectiveness of such drugs in suppressing senescent cells (Chaib et al., 2022). Cellular senescence is a valuable marker for aging as it plays a significant role in the aging of an individual. However, senescence can my caused by multiple factors unrelated to age. This makes it difficult for it to be an accurate measure of biological age as it has a high chance of being misinterpreted.

### 4.4 Epigenetic aging

Epigenetics was defined by Jaenish and Bird in 2003 as reversible heritable mechanisms that occur without alterations in the DNA sequence (Jaenisch and Bird, 2003). Epigenetic alterations have been reported to be crucial in aging and age-related diseases (Saul and Kosinsky, 2021). Epigenetic clocks are a set of CpG sites whose DNA methylation levels measure the subject age. They are highly accurate molecular chronological age correlates in humans and other vertebrates (Bell et al., 2019). This measure of biological aging is the most robust of all the other biomarkers. DNA methylation-related aging has been successfully linked to cancers, cardiovascular disorders, frailty, and mortality (Chen et al., 2022). As studied by Horvath et al., hypermethylation of CpG islands within promoters leads to transcriptional suppression, whereas hypomethylation allows gene expression. Reduction in DNA methylation is observed during aging in multiple species, which may be linked to decreased levels of DNA methyltransferase 1 (DNMT1) (Horvath et al., 2022). In contrast to this, Yagi et al. studied that *de novo* methylation increases with age due to the upregulation of other DNMTs, such as DNMT3A and DNMT3B, which insinuates that changes in DNA methylation patterns during aging can be a promising biomarker of aging. Epigenetic clocks have been identified as the gold standard for the measurement of chronological clocks as they have been studied in multiple models for the same, especially by Hannum and Horvath Fransquet et al. (2019). Hannum et al. clock was trained and tested using DNA isolated from blood of donors. It comprises 71 CpG selected from the Illumina 450 k array that strongly captures changes in chronological age, which is partly driven by age-related shifts in blood cell composition (Vijayakumar et al., 2022). The Horvath clock was constructed across multiple tissues, including the blood data from Hannum et al., as a potential “pan-tissue” master clock of chronological age and focused on capturing shared changes independent of tissue type. It included 353 CpGs that were present on the earlier generation Illumina 27 k array (Horvath et al., 2022). These differences in training sets led to some conflicting findings between reported associations of the Horvath clock. After the studies by Hannum and Horvath in 2013, several other epigenetic clocks have been built, including those by Weidner et al. (3-CpG clock) (Vijayakumar et al., 2022), Lin et al. (99-CpG clock) (Lin et al., 2016) and Vidal-Bralo et al. (8-CpG clock) (Vidal-Bralo et al., 2016). All these clocks were built using linear regression methods, which have become the standard for interpretable models, especially for chronological age. As these clocks are based on alterations in DNA sequence, they are also being studied as measures for biological aging.

### 4.5 Mitochondrial dysfunction

Mitochondrial dysfunction is a hallmark of aging and age-related diseases, and it contributes to the decline of cellular function and tissue integrity (Srivastava, 2017). Mitochondria contain their own genome, termed mitochondrial DNA (mtDNA), encoding 37 genes, including 13 genes coding for proteins, two genes coding for ribosomal RNAs (16S and 12S rRNAs), and 22 genes coding for transfer RNAs (Kienzle et al., 2023). ROS, primarily generated at complexes I and III of the mitochondrial respiratory chain, cause oxidative damage to mtDNA. Mitochondria produce ATP, the cell’s energy currency, through oxidative phosphorylation (Kienzle et al., 2023). The process involves the transfer of electrons from NADH and FADH2 to oxygen in the electron transport chain, which generates a proton gradient across the mitochondrial inner membrane, driving ATP synthesis by ATP synthase. Mitochondrial function declines with age, leading to reduced ATP production and cellular energy metabolism, contributing to age-related diseases’ development. Mitochondrial dysfunction can also lead to the accumulation of mtDNA mutations, which are inherited maternally and accumulate over time due to oxidative damage. These mutations can lead to respiratory chain dysfunction, impairing mitochondrial energy production and generating reactive oxygen species (ROS) (Amorim et al., 2022). ROS can cause cellular damage, leading to oxidative stress, inflammation, and aging. The accumulation of mtDNA mutations is a hallmark of aging and can contribute to the development of age-related diseases such as neurodegeneration, cardiovascular disease, and cancer (Kong et al., 2022). Evidence indicates that mitochondrial turnover is altered during muscle aging, potentially affecting mitochondrial function and leading to sarcopenia (Boengler et al., 2017). The fission and fusion balance tend to fall off with age, with fission decreasing and leading to poorer quality control for the mitochondria and reduced mitophagy (Fajardo et al., 2022). The impaired mitochondria quality through defective mitophagy and fusion/fission imbalance may contribute to a decrease in energy production with increasing age (Chakravorty et al., 2019). Somatic mitochondrial DNA mutations accumulate with age and may contribute to mitochondrial dysfunction and mitochondrial dynamics, including mitochondrial biogenesis, mitophagy, and mitochondrial fusion/fission, which are crucial in maintaining mitochondrial function and preventing mitochondrial dysfunction during aging (Lima et al., 2022). Moreover, the mitochondrial free radical theory of aging suggests that mitochondrial dysfunction is caused by an accumulation of reactive oxygen species (ROS) that damage mitochondrial DNA, proteins, and lipids (Lagouge and Larsson, 2013). Other factors, such as MDV (mitochondrial-derived vesicles) and lysosomes, can further lead to insight into aging caused by mitochondrial dysfunction. Mitochondrial dysfunction is emerging as a critical measure of biological age, offering insights into the aging process and potential avenues for intervention. Further research is needed to fully understand the mechanisms linking mitochondrial health to aging and to develop practical applications for assessing and modulating biological age.

### 4.6 Stem cell markers

Stem cells can differentiate into specialized cells and regenerate damaged tissues. These cells could revolutionize medicine by providing treatments for various diseases and conditions (Mahla, 2016). However, as we age, the function of stem cells declines, leading to a decrease in tissue regeneration and repair (Ahmed et al., 2017). One of the leading causes of stem cell aging is the accumulation of DNA damage over time. As cells divide, errors can occur in replication, leading to mutations and other genetic damage. Exposure to environmental factors such as radiation, toxins, and oxidative stress can also cause DNA damage (McNeely et al., 2020). As stem cells age, their ability to repair this damage decreases, leading to an accumulation of mutations and other types of damage that can impair their function (Ahmed et al., 2017). Another factor contributing to stem cell aging is changes in the epigenetic landscape of the cells. Epigenetic modifications are chemical changes to the DNA molecule that can affect gene expression without altering the underlying genetic code (Pouikli and Tessarz, 2022). Environmental factors can influence these modifications and can change over time. As stem cells age, they undergo changes in their epigenetic profile that can affect their ability to differentiate into specialized cells and regenerate damaged tissues (Smith et al., 2023).

The consequences of stem cell aging are significant. As stem cells decline in function, tissue regeneration, and repair become less efficient. This can lead to various age-related diseases and conditions, including osteoporosis, Alzheimer’s disease, and cardiovascular disease. Additionally, stem cell aging can contribute to cancer development, as damaged stem cells can accumulate mutations that lead to uncontrolled cell growth (Zakrzewski et al., 2019).

Aging occurs in different types of stem cells in different ways. Skin stem cells undergo aging as a factor of an accumulation of damage over time (Mi et al., 2022). Epidermal cells exhibit a decline in vascular function, remodeling of the extracellular matrix, and reduction in the role of pigment-producing melanocytes (Papaccio et al., 2022). This leads to epidermal thinning, dermal atrophy, fragility, hair whitening, collagen biosynthesis decline, and delayed wound healing. Stem cell aging occurs due to the dysfunction of various molecular pathways, such as the Ras/ Raf MAPK, JAK/ STAT, PI3K/AKt, Wnt, and P21 pathways (Al-Azab et al., 2022). Aging also impacts the structural composition of the niche of stem cells (Oh et al., 2014). Another type of stem cell affected by the overall aging of the body is hematopoietic stem cells. These cells are responsible for generating all erythrocytes and lymphocytes in the body. Upon aging, due to the accumulation of reactive oxygen species and DNA damage, there is an imbalance in the immune response (Colom Díaz et al., 2023). Intestinal stem cells also quickly respond to epigenetic changes and damage accumulation. Intestinal architecture and cell composition vary during intestinal stem cell aging by diminished regenerative capacity, decreased crypt numbers, increased crypt length and width, increased villi length, elevated numbers of cells per crypt, and fewer proliferating cells and lineage-traced Lgr5+ cell-derived clones (Verzi and Shivdasani, 2020). Stem cell aging is directly correlated to the biological aging of the body (Mi et al., 2022). They are accurate measures of biological age. Unfortunately, only a few methods that can accurately study the quantitative correlation between stem cell aging and an individual’s biological age have been understood. More study is required to understand the exact impact of the same.

## 5 Novel methods of measuring biological age

### 5.1 Microbiome assessment

The human microbiome is a complex ecosystem of microorganisms inhabiting various body parts, including bacteria, viruses, fungi, and archaea. These microorganisms are crucial in maintaining human health, including digestion, immune function, and metabolism (Ogunrinola et al., 2020). In recent years, researchers have begun to investigate the role of the microbiome in biological aging, and evidence suggests that changes in the microbiome may contribute to age-related health problems. As we age, the composition of the microbiome changes (Kim and Benayoun, 2020). There is a decrease in the diversity of the microbiome, with a shift towards a more pro-inflammatory state. This shift is associated with gut microbiome changes, which are thought to play a vital role in the aging process (Bairamian et al., 2022). The gut microbiome produces short-chain fatty acids essential for gut health and immune function. As we age, the production of these fatty acids decreases, leading to a decline in gut health and increased inflammation. The changes in the microbiome are thought to contribute to age-related health problems, including chronic inflammation, obesity, and insulin resistance (Al Bander et al., 2020). Recent studies have identified an abundance of fecal Christensenellaceae, Porphyromonadaceae, and Rikenellaceae. It is understood to be associated with more favorable body composition in old age, specifically in lower abdominal adiposity (Ratiner et al., 2022).

Changes in the gut microbiome have been associated with the development of age-related diseases such as type 2 diabetes, cardiovascular disease, and cancer. Additionally, changes in the microbiome have been linked to cognitive decline and neurodegenerative diseases such as Alzheimer’s. Studies are exploring various approaches to modulate the microbiome to promote healthy aging (Ren et al., 2023).

A common approach to microbiome enrichment is to use probiotics and prebiotics to promote the growth of beneficial microorganisms in the gut. Probiotics are live microorganisms that can provide health benefits when consumed, while prebiotics are substances that promote the development of beneficial organisms (Wang et al., 2021). Another approach is to use fecal microbiota transplantation (FMT), which involves transferring fecal matter from a healthy donor to a patient with an unhealthy microbiome (Kim and Gluck, 2019). Some microorganisms, such as *Christensenella, Akkermansia, and Bifidobacterium*, are commonly found in centenarians and are identified to indicatelife-prolonging capabilities (Wu et al., 2022).

The microbiome plays a significant role in the metabolomics of the body. Some of the primary metabolites influenced by the microbiome are SCFAs, amino acids, bile acids, vitamins, tryptamine, histamine, serotonin, dopamine, para-cresol, and phenylacetylglutamine12. They are involved in shifts in the body’s metabolic pathways (Wu et al., 2021). Some studies also suggest that many microbial metabolites can affect the activity of the liver and the endocrine system, for example, the onset of type 2 diabetes (Herrema and Niess, 2020). The microbiome is also involved in properly functioning immunomodulatory effects and neurological changes. For example, patients with IBD exhibited a reduction in the microorganisms showcasing anti-inflammatory properties, such as *Faecalibacterium prausnitzii* (Zheng et al., 2020). Research suggests an impact of the microbiome on the entric and central nervous system and a significant role in the development of age-related diseases such as Alzheimer’s and Parkinson’s (Szandruk-Bender et al., 2022). All of which still require extensive research. Although research links the microbiome to multiple physiological processes in the body, all this information is still rudimentary. Numerous factors, such as the internal and external environment, influence the microbiome. Increased frailty, medication intake, surgery, reduced physical activity, and diet quality play a significant role in the microbiome’s composition in an older individual (Ahn and Hayes, 2021). Aging individuals experience a loss of dominant common taxa (such as *Prevotella, Faecalibacterium, Eubacterium rectale, Lachnospira, Coprococcus,* and the health-associated genus *Bifidobacterium*). These taxa appeared to be replaced by a second group (such as *Akkermansia, Christensenellaceae, Butyricimonas, Odoribacter, and Butyricicoccus*) and pathobionts (such as *Eggerthella, Bilophila, Fusobacteria, Streptococcus, and Enterobacteriaceae*) (Assis et al., 2022). Studies have also associated microorganisms with healthy and unhealthy aging; healthy aging involves *Prevotella, Faecalibacterium, Eubacterium rectale, Coprococcus, Bifidobacterium,* and*Rosburia*. Unhealthy aging consists of the presence of pathobionts such as *Eggerthella, Bilophila, Desulfovibrio, fusobacterium aerotruncus, Streptococcus, Escherichia, Akkermansia, Christensenellaceae, Odoribacter, Butyricimonas, Butyrivibrio, Barnesiella, Oscillospira* (Salazar et al., 2023).

Although the impact of the microbiome on aging is significant, the need for more information about multiple ecological factors prevents it from being a gold standard measure of biological aging. Diverse studies are being conducted to understand and implement the microbiome to measure biological aging.

### 5.2 Exosomes

Exosomes have emerged as potential candidates for biological aging markers due to their cellular communication involvement and ability to reflect cells’ physiological state (D’Anca et al., 2019). They carry various molecules, including proteins, lipids, and nucleic acids, that can provide information about the cellular processes and alterations associated with aging (Zhang et al., 2019). Studies have shown that exosome content and composition change with age. For example, the levels of specific proteins or miRNAs (microRNAs) within exosomes may vary in older individuals compared to younger individuals (Liu et al., 2020). These changes in exosome cargo might reflect age-related cellular dysfunction, inflammation, oxidative stress, and other processes associated with aging. Evidence suggests that exosomes play a pivotal role in regulating aging mechanisms. They can target mRNA transcription and translation, and their cargo is influenced by the status of the parent cell and its microenvironment (Hamdan et al., 2021). Changes in the composition and function of exosomes have been observed with aging, which can impact tissue homeostasis and contribute to the progression of age-related diseases (Cheon and Lee, 2021). Exosomes have been studied in the context of age-related neurodegenerative diseases, such as motor neuron disease and Alzheimer’s disease (Anakor et al., 2021). They are involved in various processes, including exosome biogenesis and secretion, uptake mechanisms, and signaling within the central nervous system (Gurung et al., 2021). Exosomes derived from senescent cells have been found to contain proteins and microRNAs associated with senescence, which may contribute to the development of neurodegenerative diseases (Wallis et al., 2020). Exosomes can potentially serve as biomarkers for age-related diseases, including dementia. They can predict individual cognitive trajectories and may have diagnostic value. However, further research and clinical trials are needed to validate the accuracy and effectiveness of exosome-based diagnostics (He et al., 2023). Aging can alter the protein composition of exosomes. For example, Galectin-3, which plays a role in osteoblast maturation, was reduced in the plasma exosomes of elderly individuals, potentially resulting from the loss of stem cell functionality in the aging process (Weilner et al., 2016). Exosomes contain various lengths of genomic DNA fragments and are one of the major routes of DNA secretion. DNA secretion from exosomes increases with aging (Takasugi, 2018). Additionally, ncRNAs inside exosomes have been implicated in physiological aging and age-related neurodegenerative diseases (Wang et al., 2022). Exosomes creating a pro-inflammatory environment can accelerate the aging process. The cargo carried by exosomes, including inflammatory molecules, can contribute to the development and progression of age-related diseases (Sanz-Ros et al., 2022). The study of exosomes and their role in aging is still in its early stages, and further research is needed to fully understand their functions and mechanisms of action.

### 5.3 Artificial intelligence

Combined with barcoding technologies, single-cell sequencing and isolation increasingly expand the capabilities to measure biological age. The information gained from this type of sequence can be applied to multiple transcriptomic, proteomic, and metabolomic clocks. A common method to understand and predict biological age is to apply machine learning algorithms to develop aging clocks. These algorithms combine various biological aging clocks (proteomic, transcriptomic, epigenetic, and metabolomic) and extrapolate data in combination with sequencing results. These clocks comprise non-invasive data, such as MRI, facial imaging, and fundus imaging. AI and machine learning approaches combine various factors to predict an individual’s biological age. This includes molecular signatures such as DNA methylation, Transcriptome, and proteome information. Other factors include an individual’s cellular modalities, such as blood cell count, telomere attrition, and mitochondrial dysfunction. Whole organism cohort analysis can also be incorporated into the algorithms to measure biological age.

Modeling of biological age using machine learning uses various statistical methods of measurement. The earliest form of statistical analysis is linear regression, which is still used in algorithms. Over time, advanced AI models now utilize deep neural networks (DNN), including but not limited to convolutional neural networks and recurrent neural networks. These database networks consist of multiple information sections and can accurately perform multiple non-linear statistical analyses. The models to measure biological age using AI tools are ever-changing. The recent advancements in transformer systems, such as DeepMind’s Alphafold 2 and 3, GPT 3 and 4, and BERT. These tools are currently being used widely in research and clinics for diagnosis and prognosis prediction.

Multi-omics data is currently being used to train machine learning algorithms that can identify an individual’s biological age. Previously developed locks, such as DNA methylation clocks, are being used to train such models. MethylNet age prediction clock is an AI-based clock that was developed in 2020 that uses DNAmAge datasets to generate preliminary predictions. It can study cellular differences, grasp higher-order information about cancer sub-types, estimate age, and capture smoking-related factors concordantly with known differences. Other models include an AI-based neural network built on information from multiple DNA pathway datasets identified from skin biopsies. Some transformer-based models have also been designed to utilize GPT modes to run linear models and predict age using DNA methylation and transcriptome data. In 2024, a Python-based clock called Pyaging was developed. It combines an accelerated GPU with all currently available clocks to measure biological age. It also runs linear regression statistical analysis to ensure the accuracy of the predicted result.

Some other aging clocks based on the gut microbiome and plasma protein datasets have also been developed but are yet to be studied in detail. Results obtained from single-cell sequencing in combination with the above-mentioned clocks can help generate a clock that can efficiently predict biological age. The current clocks cannot predict biological age independently as they only focus on a singular aspect of aging compared to the multifactorial disease it is. Therefore, biological aging prediction using these clocks is always incomplete regarding information. A combination of these clocks efficiently predicts biological aging in multiple age groups. The constant learning ability of AI tools also improves the model’s accuracy and gives more insight into an individual’s biological age.

## 6 Concluding remarks

In conclusion, measuring biological age represents a crucial approach to understanding aging and age-related diseases at a molecular level. Advances in geriatric science have provided us with multiple methods to assess biological age, including telomere length, epigenetic clocks, cellular senescence, and mitochondrial function (Table 1). These methods are not very accurate as they are dependent on multiple external factors such as environment, lifestyle, genetics etc. Over time, these methods have become much more cost effective, but none of them can be considered the best. While no gold standard has been established yet, combining these methods can yield statistically significant measurements of biological age. To make further progress, it is essential to conduct additional research aimed at establishing criteria that link the hallmarks of aging to age-related disorders. This can involve large-scale studies with various sample types, such as blood, tissue, saliva, and biological fluids, to comprehensively understand the aging process. Moreover, the roles of the microbiome and exosomes in aging are increasingly recognized as significant. The microbiome influences various aspects of the human system and is associated with healthy and unhealthy aging. It also impacts neuronal networks and endocrine processes interconnected with aging-related processes. Similarly, exosomes play crucial roles in modulating degeneration in the neuronal system. Artificial intelligence is indicated to be one of the best ways to measure biological age as it can seamlessly combine multiple methods of measurement into one single algorithm, thereby increasing the accuracy of the prediction. A lot of work still needs to be done on developing these algorithms on machine-learning platforms.

**TABLE 1:** Measures of Biological aging.

Biological aging measure	Tissue	Advantages	Disadvantages
Telomere length	Blood	• Telomere length correlates with chronological age throughout the entire life course • Telomere length is highly species-specific, making it a suitable biomarker of human aging • Telomere length is linked to basic biology and correlates with aging and aging-related disease • Telomere length can be measured repeatedly and accurately without harming the person	• The evidence suggesting telomere length is a biomarker of aging in humans is equivocal • Telomere length has high inter-individual variability • Markers of biological aging may change over the lifespan, and a single biomarker may not be sufficient to reflect aging across a variety of biological systems • Telomere length can be affected by lifestyle factors such as diet and stress • Individual telomere tests are a poor guide to an individual’s biological age
Genomic instability	Blood	• Genomic instability is a hallmark of aging and is associated with age-related diseases • Genomic instability can be measured using various techniques, such as single nucleotide polymorphism (SNP) microarray data • Genomic instability can be used to identify individuals who are at higher risk of developing age-related diseases	• Genomic instability is a relatively small target size, which limits its ability to capture the full extent of DNA damage and mutations in cells • The accumulation of genomic abnormalities is influenced by the quality of the repair pathways, which may also decline with age • The frequency of detectable genomic abnormalities is low at birth and increases with age, but their frequency is still very low, and no effect on viability was detected • It is still unclear how much deviation of epigenetic age from chronological age is driven by different rates in biological aging or genetically determined differences
Cellular Senescence	Organ tissue	• Cellular senescence plays a beneficial role in regulating embryonic development, wound healing, and resolution of fibrosis • Senescent cells can be used as molecular biomarkers to study the role of senescent cells *in vivo* • Senescence can be used to limit tumor progression	• The prolonged presence of senescent cells can hamper tissue repair • Senescence causes a loss of tissue-repair capacity • Senescent cells can develop a characteristic pathogenic senescence-associated secretory phenotype (SASP) that drives secondary senescence and disrupts tissue homeostasis, resulting in loss of tissue repair and regeneration • Senescent cells can reduce tissue repair, increase chronic inflammation, and can even eventually raise the risk of cancer and other age-related diseases
Epigenetic Aging	Blood	• Epigenetic changes are vital to normal biological functioning and can affect natural cycles of cellular death, renewal, and senescence • Epigenetic changes have a huge influence on the aging process • Epigenetic aging is a novel measure of biological age, reflecting exposures and disease risks independent of chronological age • Epigenetic age acceleration may be a useful biomarker to estimate functional and cognitive aging among older women	• The association between epigenetic modifications and age is still not fully understood • Epigenetic aging is a complex multifactorial biological process shared by all living organisms • Epigenetic alterations represent one crucial mechanism behind the deteriorated cellular functions observed during aging and in age-related disorders • There is no single measure of biological age, and specific components of aging biology should be focused on and interrogated • Epigenetic aging and inflammation are largely independent markers of biological aging and may be used jointly to predict mortality
Mitochondrial dysfunction	Blood	• Mitochondrial dysfunction is one of the key hallmarks of aging and is linked to the development of numerous age-related pathologies including metabolic syndrome, neurodegenerative disorders, cardiovascular diseases, and cancer • There is accumulating evidence that mitochondrial respiratory malfunction is associated with aging-associated complex diseases • Mitochondrial dysfunction promotes the development and progression of metabolism- and aging-related disorders	• These observations do not necessarily imply a causal relationship between mitochondrial dysfunction and human aging • Much of the recent work has cast doubt on the mitochondrial free radical theory of aging, but at the same time, important steps forward have been made in better understanding the nature of mitochondrial aging • Progress in our understanding of these diseases has been hampered by the sensitivity and throughput of systems employed to quantify dysfunction and inherent limitations of the biological systems studied • Old age is associated with impaired mitochondrial function, and this has been proposed as a proximal mechanism for aging
Stem cell markers	Blood, organ tissue	• Stem cells are capable of self-renewal and differentiation into various cell types, making them a valuable tool for regenerative medicine • Stem cell markers can be used to identify and isolate stem cells from different tissues and organs • Stem cell markers can be used to monitor changes in stem cell populations during aging and disease progression	• Stem cell markers are not always specific to stem cells and can be expressed by other cell types • The expression of stem cell markers can vary depending on the tissue or organ being studied • Aging can affect the function and regenerative potential of stem cells, making it difficult to use stem cell markers as a reliable measure of biological aging • Stem cell markers may not be able to capture the full complexity of the aging process, which involves multiple cellular and molecular changes
Microbiome assessment	Organ tissue	• The gut microbiome changes along with physiological aging and may play a pivotal role in a variety of age-related processes • The gut microbiome lies at the core of many age-associated changes, including immune system dysregulation and susceptibility to diseases • The gut microbiota undergoes extensive changes across the lifespan, and age-related processes may influence the gut microbiota and its related metabolic alterations • Microbiome-based features have been included in aging clocks, which can be used to predict a personal status • Human microbiome aging clocks are considered a reliable way to measure the passage of time in a gut community and to distinguish two temporally distinct groups	• Microbiome differs in each individual as well as demographical, geographical, and dietary impacts constitute challenges in integrating the microbiome in personalized aging models • Other microbiome members, such as fungi, archaea, and viruses, are also potential aging biomarkers • The expression of microbiome markers can vary depending on the tissue or organ being studied • The microbiome is just one of many factors that contribute to the aging process, and its role in aging is still not fully understood • The complexity of the microbiome and its interactions with the host make it difficult to use microbiome markers as a reliable measure of biological aging
Exosomes	Blood	• Exosomes contain a variety of biomolecules, including nucleic acids, proteins, and lipids, that can be used as potential biomarkers for aging and age-related diseases • Exosomes can be isolated from various body fluids, including blood, urine, and cerebrospinal fluid, making them a non-invasive and easily accessible source of biomarkers • Exosomes can be used as therapeutic agents for age-related diseases, as they can deliver functional biomolecules to target cells • Exosomes can be used as a diagnostic tool for age-related cognitive decline, as they are more cost-effective and less invasive than currently available biomarkers	• The expression of exosome markers can vary depending on the tissue or organ being studied • The further clinical application of exosomes has been greatly restrained by the lack of high-quality separation and component analysis methods • The exact functions of exosomes are still being investigated, and more research is needed to understand their role in health and disease fully • The complexity of exosomes and their interactions with the host make it difficult to use exosome markers as a reliable measure of biological aging

By conducting in-depth research into these methods and their connections to the aging process, we have the potential to establish gold standards for biological age assessment. This could enhance our understanding of aging and pave the way for more effective interventions to promote healthy aging and mitigate age-related diseases.
